# Association study of C-reactive protein associated gene HNF1A with ischemic stroke in Chinese population

**DOI:** 10.1186/s12881-016-0313-3

**Published:** 2016-07-26

**Authors:** Haibin Shi, Song Leng, Hui Liang, Yan Zheng, Lidian Chen

**Affiliations:** 1 Fujian University of Traditional Chinese Medicine, No.1 Qiuyang Road, Shangjie, Minhou, Fuzhou, 350122 China; 2 Fujian University of Traditional Chinese Medicine Subsidiary Rehabilitation Hospital, Fuzhou, China; 3 Academy of Integrative Medicine, Fujian University of Traditional Chinese Medicine, Fuzhou, China; 4 Health Management Center, The Second Hospital of Dalian Medical University, Dalian, China; 5 Department of Neurology, The People’s Hospital of Fujian Province, Fuzhou, China; 6 The Second People’s Hospital Affiliated to Fujian University of Traditional Chinese Medicine, Fuzhou, China

**Keywords:** Ischemic stroke, C-reactive protein, HNF1A, Single nucleotide polymorphism, Association study

## Abstract

**Background:**

Ischemic stroke is a life-threatening condition due to obstructed blood supply of the brain. Elevation of plasma C-reactive protein, an important inflammatory marker, was known to associate with increased risk of ischemic stroke. Previous studies reported association between genetic variants of HNF1A and plasma level of C-reactive protein. The HNF1A gene encodes a hepatocyte transcription factor which might have regulatory effects on C-reactive protein synthesis in liver. Therefore, the C-reactive protein associated gene HNF1A seems to be a promising candidate gene for ischemic stroke.

**Results:**

We used HNF1A as a candidate gene of ischemic stroke and evaluated seven common variants of HNF1A for their contribution to ischemic stroke. The association analysis of HNF1A variants with ischemic stroke was performed in a Chinese population with 918 cases and 979 controls. For total ischemic stroke and large vessel disease subtype, none of variants exceeded significant threshold. For small vessel disease subtype of ischemic stroke, the G allele of rs7953249 showed nominal association (OR = 0.82, *p* = 0.04) after data adjustment for conventional risk factors. However, our preliminary results did not survived bonferroni correction for multiple comparisons.

**Conclusions:**

Common genetic variants of HNF1A showed nominal association with small vessel disease subtype of ischemic stroke though not survived bonferroni correction for multiple comparisons. The association between HNF1A and ischemic stroke is limited by small effects of individual SNPs. Our study provided additional genetic evidences to understand the role of HNF1A gene and C-reactive protein underlying ischemic stroke.

**Electronic supplementary material:**

The online version of this article (doi:10.1186/s12881-016-0313-3) contains supplementary material, which is available to authorized users.

## Background

Stroke accounts for the second most common cause of death in the world [1]. There were 2.5 million patients newly identified as stroke cases and 1.6 million patients died of stroke every year in China [2]. There were two clinical subtypes of stroke namely ischemic stroke and hemorrhagic stroke. In China, more than 60 % stroke patients were ischemic characterized by obstructed blood supply of the brain [3, 4]. Given the huge financial burden imposed by healthcare expenses of stroke, there have been intensive efforts to develop effective intervention and prevention strategies against this life-threatening disease [5, 6]. Previous epidemiological studies of ischemic stroke proposed several risk factors including hypertension, cigarette smoking, diabetes and obesity [7]. In addition to previously proposed conventional risk factors, genetic factors were also suggested to play a role underlying ischemic stroke [8].

Some ischemic stroke cases could be attributed to mendelian disorders which are caused by mutation of a single gene [9]. Whereas, genetic etiologies underlying most ischemic stroke cases were believed to be polygenic [10]. The candidate gene approach has been widely applied to identify susceptibility genes of ischemic stroke upon previous biological and functional understanding of the disease. Atherosclerosis has been known as a common cause of ischemic stroke [11]. It was reported that inflammation might associate with plaque instability and finally result in atherosclerosis [12, 13]. Meanwhile, observational studies also found that elevation of plasma C-reactive protein (CRP), an important inflammatory marker, was associated with increased risk of ischemic stroke [14, 15]. Therefore, genes affecting CRP level seem to be promising candidate genes of ischemic stroke.

Circulating level of CRP is directly associated with the CRP gene. Indeed, genetic variants of CRP gene were identified for their contribution to both CRP level and susceptibility of ischemic stroke [16]. However, CRP gene itself could only explain a small portion of CRP variance. In addition to CRP gene, genome-wide association studies (GWAS) identified a number of genetic variants associated with circulating level of CRP. As such, the HNF1A gene encoding Hepatocyte Nuclear Factor 1 Homeobox A were found to associate with CRP [17, 18]. HNF1A is mainly expressed in liver and acts as a transcription factor. As CRP is mainly synthesized in liver by hepatocytes [19], the HNF1A gene might be an important regulator of CRP and associate with ischemic stroke through its regulatory effects on CRP.

In the present study, we selected HNF1A as a candidate gene of ischemic stroke and focused on several single nucleotide polymorphisms (SNPs) of HNF1A that were known to be associated with circulating CRP levels. We performed SNP genotyping and association analysis in a large unrelated Chinese population. The association between HNF1A and ischemic stroke and its subtypes provided further evidence to understand genetic etiologies underlying ischemic stroke.

## Methods

### Subjects

In total, 918 ischemic stroke cases and 979 healthy controls were recruited from the Second People’s Hospital Affiliated to Fujian University of Traditional Chinese Medicine, Fujian Provincial Hospital, Fuzhou General Hospital of Nanjing Military Command and Fujian University of Traditional Chinese Medicine Subsidiary Rehabilitation Hospital. Their demographic information was indicated in Table 1. This study was approved by the institutional review board of each participating hospitals. Written consents and peripheral blood samples were obtained from each participant. All participants are genetically unrelated Han Chinese. The stroke status of each ischemic stroke cases was determined by Magnetic Resonance Imaging and their clinical records as confirmed by two clinicians. The large-vessel disease (LVD) and small-vessel disease (SVD) subtype of each ischemic stroke case was determined by the TOAST stroke subtype classification system [20]. Healthy controls without history of ischemic stroke and neurological impairments were recruited from healthcare center of aforementioned hospitals. The demographic information of all participants was listed in Table 1.

**Table 1:** Demographic information of participants

Group	Cases (*n* = 918)	Controls (*n* = 979)
Large Vessel Disease, n (%)	465 (51 %)	/
Small Vessel Disease, n (%)	453 (49 %)	
Age, years	69.39 ± 10.45*	67.04 ± 10.26
Male, n (%)	601 (65 %)*	562 (57 %)
Female, n (%)	317 (35 %)*	417 (43 %)
Hypertension, n (%)	216 (24 %)*	316 (32 %)
Diabetes, n (%)	598 (65 %)*	126 (13 %)
Smoking, n (%)	626 (68 %)*	170 (17 %)
Drinking, n (%)	736 (80 %)*	81 (8 %)
Triglyceride, mmol/L	1.59 ± 0.94	1.60 ± 0.91
Total Cholesterol, mmol/L	4.62 ± 1.21*	5.22 ± 1.08
Low Density Lipoprotein, mmol/L	3.04 ± 1.66*	3.42 ± 1.00
High Density Lipoprotein, mmol/L	1.20 ± 0.52*	1.35 ± 0.34

Data were shown as mean ± standard deviation (SD) or as n (%). Significant differences between cases and controls were indicated with an asterisk (*)

### SNP selection and genotyping

Eight SNPs of HNF1A namely rs7310409, rs735396, rs1169300, rs2464196, rs7953249, rs2650000, rs1169302 and rs1169307 previously associated with plasma CRP were selected for our study [18]. Due to failure of primer design,rs735396 was replaced by another SNP in linkage disequilibrium namely rs1169306. In addition, rs1169300 was excluded as it was in linkage disequilibrium with rs2464196. Finally, there were seven SNPs of HNF1A namely rs1169302, rs1169306, rs1169307, rs2464196, rs2650000, rs7310409 and rs7953249 selected for subsequent genotyping and association analysis.

Genotyping were performed at CapitalBio Corporation (Beijing, China) with Sequenom MassARRAY platform (San Diego, U.S) according to the manufacturer’s protocol. Briefly, genomic DNA was extracted from whole blood of each individual using Wizard® Genomic DNA Purification Kit (Promega, Madison, WI, USA). DNA concentration was determined by NanoDrop 1000 (Waltham, U.S). Multiplex reaction primers were designed using the MassARRAY Assay Design software package (v3.1). Mass determination was carried out with the MALDI-TOF mass spectrometer and Mass ARRAY Type 4.0 software was used for data acquisition.

### Statistical analysis

Each SNP was tested for Hardy-Weinberg equilibrium (HWE). Associations between HNF1A and ischemic stroke and its subtypes were analyzed under different models (additive, dominant, recessive and genotype) through PLINK software [21]. The odds ratio (OR) and its corresponding 95 % confidence interval (L96 and U95) were used to indicate the effect size of each variants. The association results were adjusted for known risk factors of ischemic stroke including age, sex, hypertension, diabetes, smoking, drinking, triglyceride, total cholesterol, low density lipoprotein and high density lipoprotein.

## Results

In this study, we genotyped seven common SNPs of HNF1A in a large unrelated Chinese population and performed case–control based association analysis with ischemic stroke and its subtypes. The genotype distribution of each SNP in case and control groups was analyzed under additive, dominant, recessive and genotype models for its association with ischemic stroke and its subtypes. Our results were adjusted for known risk factors of ischemic stroke including age, sex, hypertension, diabetes, smoking, drinking, triglyceride, total cholesterol, low density lipoprotein and high density lipoprotein.

The association between seven SNPs of HNF1A and overall ischemic stroke were indicated in Table 2. Before data adjustment, none of these SNPs showed significant association with overall ischemic stroke (minimum *p* = 0.09). After data adjustment for known risk factors, none of above results became significant (minimum *p* = 0.16).

**Table 2:** Association analysis of HNF1A with overall ischemic stroke

SNP	Allele	Model	Unadjusted				Adjusted			
			OR	L95	U95	*p*-value	OR	L95	U95	*p*-value
rs1169302	T	Additive	0.97	0.84	1.12	0.67	0.93	0.78	1.10	0.37
		Dominant	0.94	0.79	1.13	0.54	0.88	0.71	1.10	0.27
		Recessive	1.02	0.74	1.40	0.91	0.98	0.67	1.44	0.93
		Genotype TT	0.99	0.71	1.37	0.94	0.93	0.62	1.37	0.70
		Genotype TG	0.94	0.77	1.13	0.49	0.88	0.70	1.10	0.26
rs1169306	C	Additive	1.03	0.91	1.17	0.65	1.00	0.85	1.17	0.99
		Dominant	0.98	0.79	1.21	0.86	0.95	0.73	1.22	0.67
		Recessive	1.10	0.89	1.37	0.36	1.14	0.88	1.47	0.32
		Genotype CC	1.06	0.82	1.38	0.65	1.00	0.73	1.37	0.99
		Genotype CT	0.94	0.75	1.18	0.62	0.83	0.63	1.08	0.16
rs1169307	T	Additive	0.95	0.79	1.15	0.61	0.95	0.75	1.20	0.69
		Dominant	0.94	0.76	1.17	0.59	0.95	0.73	1.22	0.67
		Recessive	0.98	0.47	2.04	0.96	0.98	0.39	2.48	0.97
		Genotype TT	0.97	0.46	2.02	0.93	0.97	0.38	2.45	0.94
		Genotype TC	0.94	0.76	1.17	0.59	0.94	0.73	1.23	0.67
rs2464196	G	Additive	1.04	0.91	1.18	0.58	1.01	0.86	1.18	0.89
		Dominant	0.99	0.80	1.22	0.90	0.89	0.69	1.15	0.37
		Recessive	1.12	0.90	1.39	0.30	1.16	0.90	1.50	0.26
		Genotype GG	1.08	0.83	1.40	0.58	1.03	0.75	1.40	0.87
		Genotype GA	0.94	0.76	1.18	0.62	0.83	0.64	1.09	0.18
rs2650000	A	Additive	0.90	0.79	1.02	0.09	0.91	0.78	1.07	0.26
		Dominant	0.84	0.68	1.03	0.09	0.86	0.67	1.11	0.24
		Recessive	0.89	0.72	1.10	0.28	0.91	0.71	1.18	0.49
		Genotype AA	0.80	0.61	1.04	0.09	0.83	0.61	1.14	0.26
		Genotype AC	0.85	0.68	1.07	0.16	0.87	0.67	1.14	0.32
rs7310409	A	Additive	0.97	0.85	1.11	0.69	1.01	0.86	1.18	0.94
		Dominant	0.97	0.80	1.17	0.75	1.00	0.79	1.26	0.98
		Recessive	0.96	0.75	1.23	0.74	1.03	0.76	1.37	0.87
		Genotype AA	0.95	0.72	1.24	0.69	1.02	0.73	1.42	0.91
		Genotype AG	0.98	0.80	1.20	0.82	0.99	0.77	1.27	0.93
rs7953249	G	Additive	0.90	0.79	1.03	0.12	0.91	0.77	1.06	0.22
		Dominant	0.84	0.68	1.03	0.10	0.85	0.66	1.09	0.21
		Recessive	0.90	0.72	1.13	0.36	0.91	0.70	1.19	0.48
		Genotype GG	0.81	0.62	1.06	0.12	0.82	0.60	1.13	0.23
		Genotype GA	0.85	0.69	1.06	0.16	0.86	0.66	1.12	0.27

All SNPs were analyzed under allele, genotype, dominant and recessive models. Statistical analysis was performed using Chi-square test. *P*-value less than 0.05 were indicated in bold. Effect size was indicated in odds ratio (OR) with 95 % confidence interval (L95 and U95). After bonferroni correction, none of the associations highlighted in bold remained significant

The association between seven SNPs of HNF1A and large vessel disease subtype of ischemic stroke were indicated in Table 3. Before data adjustment, none of these SNPs showed significant association with large vessel disease subtype of ischemic stroke (minimum *p* = 0.25). After data adjustment for known risk factors, the T allele of rs1169302 showed marginal association under dominant model (TT + TG v.s GG, OR = 0.88, *p* = 0.05). In addition, its heterozygote genotype TG also showed marginal association (OR = 0.87, *p* = 0.05). After bonferroni correction for multiple comparisons, none of these seven SNPs exceeded significant threshold.

**Table 3:** Association analysis of HNF1A with large vessel disease

SNP	Allele	Model	Unadjusted				Adjusted			
			OR	L95	U95	*p*-value	OR	L95	U95	*p*-value
rs1169302	T	Additive	0.92	0.77	1.10	0.35	0.84	0.68	1.04	0.11
		Dominant	0.88	0.70	1.10	0.26	0.76	0.58	1.00	0.05
		Recessive	0.98	0.66	1.45	0.91	0.94	0.58	1.50	0.78
		Genotype TT	0.92	0.61	1.38	0.69	0.82	0.50	1.34	0.44
		Genotype TG	0.87	0.69	1.10	0.25	0.75	0.56	1.00	0.05
rs1169306	C	Additive	1.01	0.87	1.19	0.86	0.96	0.79	1.17	0.72
		Dominant	1.00	0.77	1.29	0.98	0.84	0.61	1.14	0.26
		Recessive	1.04	0.80	1.36	0.75	1.09	0.80	1.50	0.58
		Genotype CC	1.03	0.75	1.42	0.86	0.94	0.64	1.38	0.74
		Genotype CT	0.98	0.75	1.29	0.89	0.79	0.57	1.10	0.17
rs1169307	T	Additive	0.95	0.75	1.20	0.67	0.98	0.73	1.30	0.87
		Dominant	0.92	0.71	1.19	0.52	0.96	0.70	1.31	0.78
		Recessive	1.26	0.55	2.90	0.59	1.18	0.41	3.44	0.76
		Genotype TT	1.23	0.53	2.83	0.63	1.17	0.40	3.40	0.78
		Genotype TC	0.90	0.68	1.18	0.43	0.94	0.68	1.31	0.72
rs2464196	G	Additive	1.03	0.87	1.20	0.76	0.98	0.81	1.19	0.85
		Dominant	1.01	0.77	1.30	0.97	0.85	0.62	1.17	0.32
		Recessive	1.07	0.82	1.39	0.64	1.12	0.82	1.54	0.48
		Genotype GG	1.05	0.76	1.45	0.75	0.97	0.66	1.43	0.87
		Genotype GA	0.98	0.75	1.30	0.91	0.80	0.58	1.12	0.20
rs2650000	A	Additive	1.04	0.89	1.22	0.60	1.00	0.82	1.22	0.99
		Dominant	1.06	0.81	1.38	0.67	0.97	0.71	1.34	0.87
		Recessive	1.06	0.82	1.38	0.66	1.03	0.75	1.42	0.85
		Genotype AA	1.09	0.79	1.51	0.59	1.00	0.68	1.48	0.98
		Genotype AC	1.04	0.79	1.38	0.76	0.96	0.69	1.34	0.82
rs7310409	A	Additive	1.07	0.91	1.26	0.40	1.15	0.95	1.41	0.15
		Dominant	1.09	0.85	1.38	0.50	1.14	0.85	1.53	0.38
		Recessive	1.11	0.83	1.49	0.47	1.31	0.92	1.86	0.13
		Genotype AA	1.16	0.83	1.61	0.39	1.36	0.91	2.03	0.13
		Genotype AG	1.06	0.82	1.37	0.64	1.07	0.79	1.46	0.67
rs7953249	G	Additive	0.97	0.83	1.14	0.71	1.00	0.82	1.22	0.98
		Dominant	0.94	0.73	1.22	0.66	0.96	0.70	1.32	0.81
		Recessive	0.98	0.75	1.28	0.86	1.05	0.76	1.45	0.78
		Genotype GG	0.94	0.68	1.30	0.71	1.01	0.68	1.49	0.97
		Genotype GA	0.95	0.72	1.24	0.69	0.94	0.68	1.31	0.73

All SNPs were analyzed under allele, genotype, dominant and recessive models. Statistical analysis was performed using Chi-square test. *P*-value less than 0.05 were indicated in bold. Effect size was indicated in odds ratio (OR) with 95 % confidence interval (L95 and U95). After bonferroni correction, none of the associations highlighted in bold remained significant

The association between seven SNPs of HNF1A and small vessel disease subtype of ischemic stroke were indicated in Table 4. Before data adjustment, the A allele of rs2650000 showed significant association with small vessel disease subtype of ischemic stroke under additive model (OR = 0.83, *p* = 0.03) and dominant model (AA + AC v.s CC, OR = 0.74, *p* = 0.02). Its homozygote genotype AA also showed significant association (OR = 0.70, *p* = 0.03). In addition, the G allele of rs7953249 showed significant association with small vessel disease subtype of ischemic stroke under additive (OR = 0.84, *p* = 0.03) and dominant models (GG + GA v.s AA, OR = 0.75, *p* = 0.02). Its homozygote genotype AA also showed significant association (OR = 0.70, *p* = 0.03). After data adjustment for known risk factors, only the G allele of rs7953249 remained significant under additive model (OR = 0.82, *p* = 0.04) and its homozygote genotype GG (OR = 0.66, *p* = 0.04) respectively. After bonferroni correction for multiple comparisons, none of these seven SNPs exceeded significant threshold.

**Table 4:** Association analysis of HNF1A with small vessel disease

SNP	Allele	Model	Unadjusted				Adjusted			
			OR	L95	U95	*p*-value	OR	L95	U95	*p*-value
rs1169302	T	Additive	1.02	0.86	1.21	0.81	0.99	0.81	1.22	0.95
		Dominant	1.02	0.81	1.27	0.89	1.01	0.77	1.32	0.95
		Recessive	1.06	0.72	1.56	0.77	0.94	0.60	1.50	0.81
		Genotype TT	1.06	0.71	1.59	0.77	0.95	0.59	1.54	0.85
		Genotype TG	1.01	0.79	1.28	0.96	1.02	0.77	1.36	0.89
rs1169306	C	Additive	1.05	0.89	1.23	0.57	1.05	0.86	1.27	0.63
		Dominant	0.97	0.74	1.25	0.80	0.96	0.70	1.32	0.81
		Recessive	1.17	0.90	1.52	0.24	1.18	0.86	1.61	0.31
		Genotype CC	1.10	0.80	1.51	0.57	1.10	0.75	1.61	0.63
		Genotype CT	0.91	0.69	1.20	0.49	0.90	0.65	1.26	0.54
rs1169307	T	Additive	0.95	0.75	1.21	0.70	0.90	0.67	1.21	0.50
		Dominant	0.97	0.75	1.26	0.81	0.91	0.66	1.25	0.56
		Recessive	0.70	0.25	1.94	0.49	0.69	0.19	2.50	0.58
		Genotype TT	0.70	0.25	1.94	0.49	0.68	0.19	2.45	0.56
		Genotype TC	0.99	0.76	1.29	0.92	0.92	0.67	1.27	0.63
rs2464196	G	Additive	1.05	0.89	1.23	0.56	1.05	0.87	1.28	0.59
		Dominant	0.97	0.74	1.25	0.80	0.97	0.71	1.33	0.87
		Recessive	1.18	0.91	1.53	0.22	1.18	0.86	1.62	0.30
		Genotype GG	1.10	0.80	1.52	0.55	1.11	0.76	1.63	0.59
		Genotype GA	0.91	0.69	1.20	0.49	0.91	0.65	1.27	0.59
rs2650000	A	Additive	**0.83**	**0.71**	**0.98**	**0.03**	0.84	0.69	1.02	0.07
		Dominant	**0.74**	**0.58**	**0.96**	**0.02**	0.79	0.58	1.08	0.14
		Recessive	0.83	0.63	1.09	0.18	0.79	0.57	1.09	0.15
		Genotype AA	**0.70**	**0.50**	**0.96**	**0.03**	0.70	0.47	1.04	0.08
		Genotype AC	0.77	0.58	1.00	0.05	0.84	0.61	1.16	0.29
rs7310409	A	Additive	0.88	0.75	1.04	0.14	0.90	0.74	1.10	0.30
		Dominant	0.87	0.68	1.10	0.23	0.94	0.70	1.24	0.65
		Recessive	0.81	0.59	1.11	0.20	0.77	0.53	1.13	0.18
		Genotype AA	0.76	0.54	1.08	0.13	0.77	0.50	1.17	0.22
		Genotype AG	0.90	0.70	1.15	0.41	0.99	0.74	1.34	0.96
rs7953249	G	Additive	**0.84**	**0.71**	**0.98**	**0.03**	**0.82**	**0.67**	**0.99**	**0.04**
		Dominant	**0.75**	**0.58**	**0.96**	**0.02**	0.78	0.57	1.05	0.10
		Recessive	0.83	0.62	1.10	0.18	0.75	0.53	1.05	0.09
		Genotype GG	**0.70**	**0.50**	**0.97**	**0.03**	**0.66**	**0.45**	**0.98**	**0.04**
		Genotype GA	0.77	0.59	1.01	0.06	0.83	0.60	1.14	0.25

All SNPs were analyzed under allele, genotype, dominant and recessive models. Statistical analysis was performed using Chi-square test. *P*-value less than 0.05 were indicated in bold. Effect size was indicated in odds ratio (OR) with 95 % confidence interval (L95 and U95). After bonferroni correction, none of the associations highlighted in bold remained significant

## Discussion

Although atherosclerosis was known to be a common cause of ischemic stroke, molecular pathogenesis underlying atherosclerosis and ischemic stroke remain complex and elusive. Inflammation is a series of physiological responses to stimulations of various pathogens. It occurs systemically and associates with a number of human diseases. CRP is a well-known inflammatory marker that could be quantitated in circulating blood. Elevation of circulating CRP has been recognized as a predictive marker for atherosclerosis and cardiovascular diseases. In recent years, studies in general populations observed association of CRP with ischemic stroke [15, 22, 23]. Whereas, contradictory results were also reported by studies in different cohorts [24, 25]. Identification of CRP-associated genetic variants provided an alternative aspect to elucidate contribution of CRP to ischemic stroke. Genetics variants of CRP gene which were directly associated with circulating CRP level showed significant association with ischemic stroke in a Chinese population [16]. In addition to CRP gene, circulating CRP level was associated with some regulatory genes that could also be considered as candidate genes of ischemic stroke. CRP is mainly synthesized in liver [19]. The hepatocyte nuclear factor HNF1A is a transcription factor that was believed to have regulatory effects on CRP. Indeed, association between common SNPs of HNF1A and circulating CRP level were reported in large-scale GWAS [17, 18].

In our study, we used these variants of HNF1A as candidate variants of ischemic stroke and performed association analysis in a Chinese cohort. For overall ischemic stroke and its LVD subtype, none of SNPs exceeded significant threshold. For SVD subtype of ischemic stroke, the A allele of rs2650000 and the G allele of rs7953249 showed nominal association with the disease. Given the broad effects of inflammation, interplay between CRP and conventional risk factors of ischemic stroke would cause confounding biases which might explain the inconclusive and controversial role of CRP underlying ischemic stroke in previous studies. To reduce confounding biases, we adjusted both non-modifiable risk factors (age and sex) and conventional risk factors (hypertension, diabetes, and etc.) during association analysis between HNF1A and ischemic stroke. After data adjustment, only the G allele of rs7953249 remained significant with protective effect on SVD subtype of ischemic stroke. Thus, our preliminary results further supported the independent contribution of HNF1A and CRP to ischemic stroke. In addition, we applied bonferroni correction which was known to be one of the most stringent methods for multiple comparisons to the seven SNPs of HNF1A. The significant threshold after bonferroni correction became approximate 0.007 (0.05/7) therefore none of analyzed variants persisted significant.

For polygenic diseases such as ischemic stroke, there were considerable portion of unexplained heritability remained as missing heritability [26]. Although some genetic variants were identified by candidate gene based approach, they were usually believed to be common variant with small effect [27]. In the present study, effects of each SNPs of HNF1A on ischemic stroke were limited which might explain that none of SNPs exceed the significant threshold yielded by bonferroni correction. As such, expanding sample size could be an option to confirm the association between HNF1A and ischemic stroke. Alternatively, functional characterization of HNF1A variants would be a more direct way to explain its contribution to ischemic stroke. The rs7953249, located upstream of HNF1A, was associated with plasma N-glycan levels and the HNF1A gene was shown to be a regulator of protein glycosylation [28]. Dysregulation of glycosylation was known to associate with a wide range of diseases including cancer, diabetes and cardiovascular diseases [29]. A number of mutations of HNF1A gene were associated with maturity-onset diabetes of the young (MODY), an early onset form of type 2 diabetes [30, 31]. Thus, pleiotropic effects of HNF1A variants on CRP and N-glycan are worthwhile to be further investigated.

## Conclusion

In conclusion, our study used HNF1A as a candidate gene of ischemic stroke and genotyped seven common SNPs of HNF1A in a Chinese population. We found nominal association between HNF1A and small vessel disease subtype of ischemic stroke though not survived bonferroni correction for multiple comparisons. Currently, the contribution of seven candidate variants of HNF1A to ischemic stroke should be interpreted with caution. It is worthwhile to replicate the association between HNF1A and ischemic stroke in expanded cohorts and to characterize the functional role of HNF1A underlying ischemic stroke.

## Abbreviations

CRP, C-reactive protein; GWAS, Genome-Wide Association Study; HNF1A, hepatocyte nuclear factor 1 homeobox A; HWE, Hardy-Weinberg equilibrium; LVD, large vessel disease; OR, odds ratio; SNP, single nucleotide polymorphism; SVD, small vessel disease

## Additional file

Additional file 1: Table S1. Genotype distribution of overall ischemic stroke. **Table S2.** Genotype distribution of large vessel disease. **Table S3.** Genotype distribution of small vessel disease. (DOCX 17 kb)
