# Supplementary material for: Association study of C-reactive protein associated gene HNF1A with ischemic stroke in Chinese population
Source: BMC Med Genet. 2016 Jul 26;17:51. doi: 10.1186/s12881-016-0313-3 (PMC4962403; doi:10.1186/s12881-016-0313-3)
Supplement: Additional file 1: Table S1. — Genotype distribution of overall ischemic stroke. Table S2. Genotype distribution of large vessel disease. Table S3. Genotype distribution of small vessel disease. (DOCX 17 kb) [file 12881_2016_313_MOESM1_ESM.docx]

Supplement Table S1. Genotype distribution of overall ischemic stroke

| Overall IS | | | | |
| --- | --- | --- | --- | --- |
| SNP | A1 | A2 | Cases (A1A1//A1A1//A2A2) | Controls (A1A1//A1A1//A2A2) |
| rs1169302 | T | G | 82//367//456 | 85//402//467 |
| rs1169306 | C | T | 225//437//222 | 225//492//236 |
| rs1169307 | T | C | 14//199//697 | 15//219//722 |
| rs2464196 | G | A | 224//438//221 | 222//495//236 |
| rs2650000 | A | C | 200//447//236 | 237//495//223 |
| rs7310409 | A | G | 146//454//303 | 160//482//314 |
| rs7953249 | G | A | 184//451//250 | 215//501//237 |

Supplement Table S2. Genotype distribution of large vessel disease

| LVD | | | | |
| --- | --- | --- | --- | --- |
| SNP | A1 | A2 | Cases (A1A1//A1A1//A2A2) | Controls (A1A1//A1A1//A2A2) |
| rs1169302 | T | G | 40//179//239 | 85//402//467 |
| rs1169306 | C | T | 109//227//111 | 225//492//236 |
| rs1169307 | T | C | 9//96//353 | 15//219//722 |
| rs2464196 | G | A | 109//227//110 | 222//495//236 |
| rs2650000 | C | A | 109//231//106 | 237//495//223 |
| rs7310409 | A | G | 83//230//141 | 160//482//314 |
| rs7953249 | G | A | 99//232//116 | 215//501//237 |

Supplement Table S3. Genotype distribution of small vessel disease

| SVD | | | | |
| --- | --- | --- | --- | --- |
| SNP | A1 | A2 | Cases (A1A1//A1A1//A2A2) | Controls (A1A1//A1A1//A2A2) |
| rs1169302 | T | G | 42//188//217 | 85//402//467 |
| rs1169306 | C | T | 116//210//111 | 225//492//236 |
| rs1169307 | T | C | 5//103//344 | 15//219//722 |
| rs2464196 | G | A | 115//211//111 | 222//495//236 |
| rs2650000 | A | C | 94//216//127 | 237//495//223 |
| rs7310409 | A | G | 63//224//162 | 160//482//314 |
| rs7953249 | G | A | 85//219//134 | 215//501//237 |
